# Genome-Wide Linkage Analysis Identifies a Novel Locus for Grip Strength: The Long Life Family Study

**DOI:** 10.1093/geroni/igaa057.466

**Published:** 2020-12-16

**Authors:** Adam Santanasto, Mary Wojczynski, Ryan Cvejkus, Bharat Thygarajan, Kaare Christensen, Joseph Zmuda

**Affiliations:** 1 University of Pittsburgh, PITTSBURGH, Pennsylvania, United States; 2 Washington University School of Medicine, St Louis, Missouri, United States; 3 University of Pittsburgh, Pittsburgh, Pennsylvania, United States; 4 University of Minnesota, Minneapolis, Minnesota, United States; 5 University of Southern Denmark, Odense C, Syddanmark, Denmark

## Abstract

Grip strength declines with aging, is an indicator of overall health, and predicts mortality among older adults. Herein, we quantified the genetic contributions to grip strength among 4534 individuals, belonging to 574 families in the Long Life Family Study (age 70.3 ± 15.7, range 24–110 years; 56% women). Grip strength was measured using a handheld dynamometer, and the maximum value of two trials in the stronger hand was used. Quantitative trait linkage analysis was completed using pedigree-based maximum-likelihood methods with logarithm of the odds (LOD) scores >3.0 indicating genome-wide significance. Linkage analysis in the top 10% of families contributing to LOD scores was also performed to allow for heterogeneity among families (HLOD). All analyses were adjusted for age, sex, height and field center. Grip strength was lower per one year of older age (β: -0.34 ± 0.01kg, p <0.01), and overall: 24.3% of men and 19.3% of women had “low” grip strength according to European Working Group on Sarcopenia definitions. Grip strength was highly heritable (h2 = 0.37, p<0.05). We identified a potentially novel locus for grip strength on chromosome 18p (LOD 3.18) with 26 families contributing to this linkage peak (HLOD = 10.94). Deep sequencing of the chromosome 18 region may yield fundamental insight on the biology of muscle weakness with aging, and may help identify novel therapeutic targets for treatment and prevention of this common condition.

